# Physiotherapy Screening for Referral of a Patient with Patent Foramen Ovale Presenting with Neck Pain as Primary Complaint: A Case Report

**DOI:** 10.3390/healthcare11081165

**Published:** 2023-04-18

**Authors:** Giovanni Lopez, Fabio Cataldi, Giuseppe Bellin, James Dunning, César Fernández-de-las-Peñas, Erasmo Galeno, Roberto Meroni, Filippo Maselli, Firas Mourad

**Affiliations:** 1 Department of Human Neurosciences, Sapienza University of Rome, 00185 Rome, Italy; 2 Kinesis, Department of Physiotherapy, 70126 Bari, Italy; 3 Manual Therapy Laboratory—MTLab, Department of Physiotherapy, 70123 Bari, Italy; 4 Centro Diagnostico Veneto, Department of Physical Therapy, 36030 Vicenza, Italy; 5 American Academy of Manipulative Therapy Fellowship in Orthopaedic Manual Physical Therapy, Montgomery, AL 36104, USA; 6 Montgomery Osteopractic Physical Therapy & Acupuncture Clinic, Montgomery, AL 36104, USA; 7 Department of Physical Therapy, Occupational Therapy, Rehabilitation and Physical Medicine, Universidad Rey Juan Carlos, 28922 Alcorcón, Spain; 8 Cátedra de Clínica, Investigación y Docencia en Fisioterapia, Terapia Manual, Punción Seca y Ejercicio, Universidad Rey Juan Carlos, 28922 Alcorcón, Spain; 9 Department of Physiotherapy, LUNEX International University of Health, Exercise and Sports, 4671 Differdange, Luxembourg; 10 Luxembourg Health & Sport Sciences Research Institute A.s.b.l., 50, Avenue du Parc des Sports, 4671 Differdange, Luxembourg

**Keywords:** differential diagnosis, cardiovascular diseases, heart disease risk factors, masked hypertension, white coat hypertension, hypertension

## Abstract

Neck pain is a common musculoskeletal disorder encountered by physiotherapists. However, it may be the early manifestation of more alarming conditions, such as cardiovascular diseases mimicking musculoskeletal pain. Patent foramen ovale (PFO) is a congenital heart defect consisting of a small opening between the right and the left atrium. A 56-year-old male presented with neck pain and head heaviness as primary complaints. The cardiovascular profile and the behavioral symptoms led the physiotherapist to find an exaggerated blood pressure response during exercise; in addition to subtle neurological signs, this prompted the physiotherapist to make an urgent referral. At the emergency department a PFO was diagnosed. To the best of the authors’ knowledge, this is the first case to describe a rare clinical presentation of a PFO presenting neck pain as primary complaint. This case report emphasizes the importance for physiotherapists to be able to triage patients for conditions outside their scope suggestive of further medical investigation.

## 1. Introduction

Neck pain (NP) is one of the most common musculoskeletal disorders encountered in the physiotherapy practice [1,2,3]. The incidence of NP is increasing and it is estimated that up to 70% of the general population will suffer from NP during their lives [1,4]. Even if the natural history of NP is benign most of the time, it is not uncommon to encounter life-threatening conditions mimicking a musculoskeletal disorder such as congenital craniovertebral anomalies, fractures, cancer, vascular pathologies, or cardiovascular diseases [5,6,7,8]. Therefore, it is the physiotherapist’s responsibility to triage those patients presenting with warning signs or symptoms of serious pathologies presenting as NP. The International Federation of Orthopaedic Manipulative Physical Therapists recently released a Cervical Framework confirming that differential diagnosis is essential for a safe physiotherapy practice [9]. However, the screening for the referral process for serious pathologies of the neck is a real challenge [2,4,10,11]. That is, physiotherapists should be trained to triage vascular and neurological disorders [12,13,14,15,16], including the examination of cranial nerves [16,17] and cardiovascular parameters [12,14,18,19], in order to recognize those patients’ presentations which require referral for further medical opinion [15,20,21,22].

For an early identification of cardiovascular diseases (e.g., blood flow limitation [23,24,25] and myocardial infarction [26,27]), the American Physical Therapy Association clinical practice guidelines recommend including blood pressure (BP) and heart rate (HR) measurements during the patient’s evaluation [28,29,30,31,32,33]. Symptoms of heart diseases (e.g., myocardial infarction) may present as viscerosomatic referred pain and masquerade as musculoskeletal disorders (most commonly chest/thoracic pain associated with arm and NP) [34,35,36], which are even undiagnosed in the emergency department [37]. Among these, patent foramen ovale (PFO) is the most common congenital heart defect and has been observed in almost one of every four adults [38]. Notably, PFO is usually asymptomatic until adulthood without presenting any signs or symptoms directly from PFO. However, it may cause paradoxical embolism, manifesting as stroke or visceral/peripheral ischemia [39]; PFO is strongly related to cryptogenic stroke, particularly in younger adults (<55-years-old) [40]. People with PFO may also experience hypoxemia, platypnea–orthodeoxia syndrome (which includes shortness of breath while upright along with low blood oxygen levels), and myocardial infarction, including chest/thoracic pain (notably, not experienced by about half of people) and NP [39,41]. 

To the best of the authors’ knowledge, our case report is the first to describe a subject with PFO presenting in primary physiotherapy with NP as primary complaint, highlighting the importance to include vascular pathologies in the hypothesis generation phase during the physiotherapy consultation. Importantly, delayed recognition of serious conditions mimicking or presenting with NP are not uncommon [11] and may cause fatal outcomes [15]. Therefore, the purpose of our case report is to inform clinicians of an unusual clinical presentation of a congenital heart defect potentially presented as NP and to describe the triage process that raises the index of suspicion of a patient outside the physiotherapy scope. Our case report follows the CARE checklist [42].

## 2. Case Presentation

### 2.1. Patient Information

**Figure 1:**
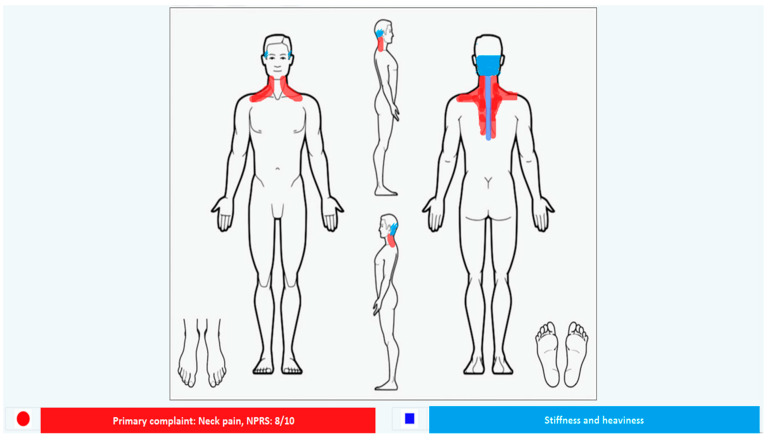
Symptoms at first assessment. In red: NP NPRS 8/10 during working and training activities; in blue: neck stiffness NPRS 7/10 and feeling of heaviness in the head.

A 56-year-old male bodybuilder and gym instructor (used to train 5 times per week), was self-referred to our physiotherapist outpatient clinic seeking help because of concerns with the primary symptom of NP with feeling of heaviness in the head. The symptoms started gradually one month before during his usual weightlifting (e.g., bench press, shoulder press, squat) and treadmill training, with NP accompanied by stiffness and heaviness to the head. Although symptoms were progressively becoming worse, being aggravated during his weightlifting legs’ training sessions (e.g., squats), he was still training. His resting baseline pain was 5/10 on a Numeric Rating Pain Scale (NPRS) (0, no pain; 10 worst pain) [43] and his most severe pain in the past 24 h was 8/10. Since the onset, he noticed limited neck rotation on both sides, with an increase of stiffness and pain during cold exposure (Figure 1).

In the previous two days the symptoms were marked upon awakening, but gradually improved after a few hours during the morning, leaving the head heaviness which impeded him to train normally. The subject had not previously visited a medical physician or physiotherapist; however, he admitted to controlling his symptoms by self-massaging his neck and shoulder region with a foam roller, avoiding any medication intake. He denied any previous trauma, night pain and dizziness.

From adolescence, he was diagnosed having ‘hyperreflexia and dysmetria of the left upper limb’ because of difficulties in the fine and coordination motor skills. In addition, he reported a surgery for testicular torsion in 1982 and a left shoulder total arthroplasty in 2018. Importantly, he and his brother have had thrombocytopenia and leukopenia [44,45,46] from a young age; his father suffers from hypertension [47]. The subject was strongly concerned and distressed because of the COVID-19 pandemic restrictions that were affecting his job [48].

### 2.2. Clinical Findings

There was observable muscle spasm on both the trapezius muscles; neck rotational mobility was limited and painful (NPRS 8/10); neck extension increased head heaviness [49]. As the history revealed an emerging pattern of cardiovascular risk factors (i.e., thrombocytopenia and leukopenia since he was young, together with hypertension familiarity and symptoms were behavior related to physical exertions), and because of the COVID-19 pandemic, vital sign measurements were performed [50,51]. Temperature was normal, and a regular pulse oxygen saturation level of 99% and 68 bpm resting heart rate (RHR) were assessed by a pulse oximeter [52]. Resting blood pressure (RBP) was measured on both arms and the higher one was used for follow-up measurements [19,47]. The mean of the last 2 measurements of the left arm—1 measurement every 2 minutes: (1) 125/85 mmHg; (2) 135/75 mmHg—was found to be 130/80 mmHg, suggesting a high-normal BP [47]. In addition, considering the worsening effect of his familiar submaximal physical activity (especially, the treadmill and weightlifting) with the goal to detect any exaggerated BP response [53,54], BP was also measured during progressive exertion on a treadmill. As muscle contraction may increase recorded BP values, the patient was advised to relax all tension in the left upper limb during exercise; BP, rate of perceived exertion (RPE) and HR measurement were performed every 3 min [55]. The treadmill was set at a speed of 5 km/h and a 2% inclination, with the goal to detect any excessive BP changes during physical exertion or the symptoms’ occurrence [56]. RBP and RHR were used as baseline measurements before exercise [55]. A progressive worsening of the measurements was observed during the test (i.e., minute 3: HR = 91 bpm, RPE = 0 and BP = 145/85; minute 6: HR = 96 bpm, RPE = 5, BP = 160/95, and familiar NP onset; minute 9: sharp increase of NP, BP = 190/100 mmHg, and RPE = 8). Additionally, during the last part of the test a progressive unsteady gait was noticed; the test was stopped at minute 9 because of the symptoms’ worsening [55,57]. Table 1 provides all the details of BP measurements during exercise. These findings and a left blepharoptosis observed after the treadmill, increased the index of suspicion for a more serious condition [17].

### 2.3. Analysis

Although RBP measurement and vital signs were unremarkable [52], as the symptoms (both musculoskeletal and neurological) were exercise induced, an adjunctive BP measurement was performed under physical exertion. The findings associated with the cardiovascular risk profile of the patient—i.e., cardiovascular disease and family history of hypertension—were key findings that led the physiotherapist to suspect that patient’s complaints were likely of cardiovascular origin [47,52,57,58,59]. Furthermore, the observed unsteadiness and blepharoptosis was suggestive for neurological compromise in need of further examination [15,60] and urgent referral [9,61]. 

#### Follow-Up and Outcomes

The patient attended the emergency department where he was hospitalized for further investigations. At the time of the neurological examination, the neurologist objectively observed the patient’s coordination difficulties—finger to nose test—and the ataxic gait; in addition, increased left biceps and quadriceps osteotendinous reflexes and bilateral ptosis—more evident on the left side—were found. Then, the decision was made to further examine the patient condition and an ophthalmologist’s opinion was requested. The Hess–Lancaster screen test confirmed a left eye ptosis and inferior oblique muscle weakness with no diplopia.

**Figure 2:**
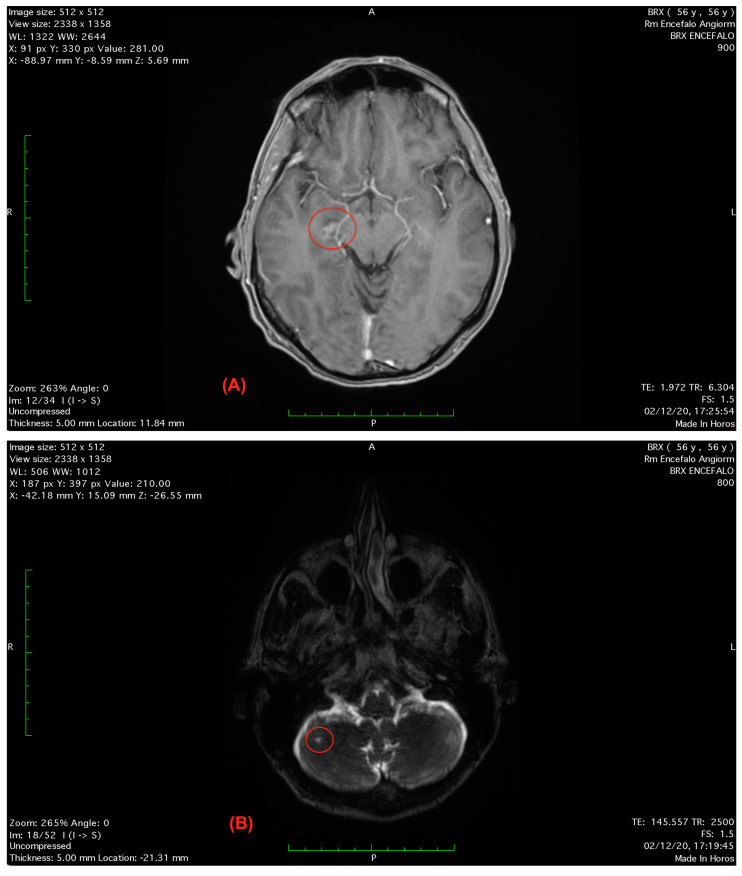
(**A**) Axial T1-weighted FLAIR scan showing a small hyper-intense areola in correspondence to the lower right midbrain’s colliculus. (**B**) Axial T2-weighted brain magnetic resonance imaging scan showing the small hyper-intense areola in the right cerebellar hemisphere.

At the following heart surgeon visit, the electrocardiography and electroencephalogram testing were unremarkable; however, the routine blood testing revealed high levels of erythrocyte sedimentation rate 15 mm/h, creatine kinase 259 U/L, leukocytes 3.65 cells/μL and erythrocytes 12.8 cells/μL. Cranial computerized tomography scans were unremarkable, while brain magnetic resonance imaging scans showed two small hyper-intense areolas in correspondence to the lower right midbrain’s colliculus and to the ipsilateral cerebellar hemisphere [62] (Figure 2A,B). 

**Figure 3:**
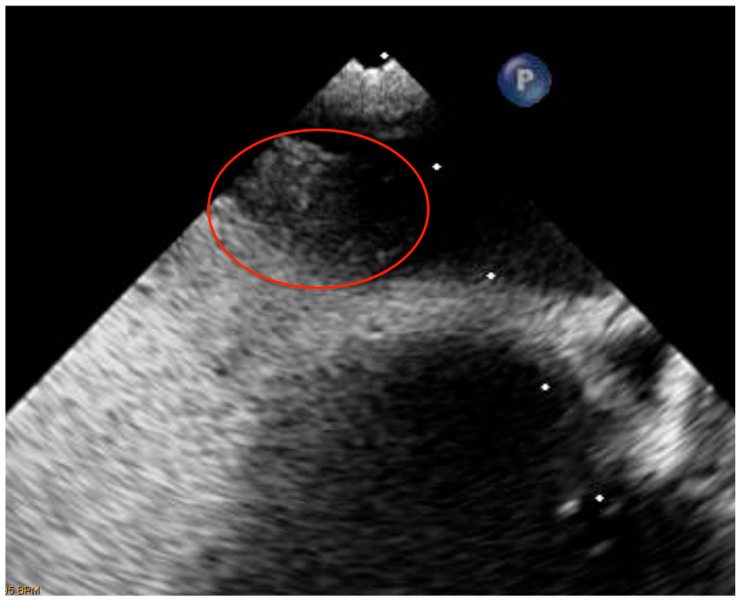
The echocardiographic image displays a secundum atrial septum defect with patent foramen ovale (PFO).

A further contrast transcranial doppler (c-TEE) and contrast transesophageal echocardiogram (c-TCD) for the study of the hemodynamics of the right-to-left shunting were set because the clinical features were suggestive of an ischemic stroke with cardio-embolic genesis (likely due to PFO) [63,64,65,66]. The c-TCD “was indicative of permanent high grade intra-cardiac shunt” and the c-TEE confirmed the existence of a “large PFO” [63,64,65,66] (Figure 3).

A diagnosis of “midbrain and right cerebellar infarction with potential cardio-embolic genesis in a subject with PFO” was made [62,67,68,69], and the patient was medicated and discharged waiting for a surgery.

Although the surgical management of PFO is still controversial, the decision was made for a surgical “right heart catheterization procedure and a percutaneous closure of the PFO from a right femoral venous access”. PFO trans-catheter closure has been shown to be the best surgical option allowing the patient to be safely discharged from hospital in 24 h [65,70]. After surgery, the patient’s symptoms immediately improved. That is, his NP drastically decreased from NPRS 8/10 to 2/10, as well as the reported head heaviness and the neurological symptoms (i.e., uncertain walking and tandem walking) improved. Thus, he was discharged with the indication for a pharmacological antibiotic and antalgic therapy (i.e., pantoprazole, acetylsalicylic acid, clopidogrel hydrogen sulfate, and cefixime) until the next cardiologic and neurologic consultations. At the first 30 day follow-up, the medication intake was changed, removing the cefixime. At the time, the patient was “feeling moderately fine and was hoping to be able to resume physical activity at high levels as before” as he was allowed by the physician to gradually return to moderate training. Therefore, he was prescribed to start with 30–40% of his previous 1 maximum repetition (1-RM represents the maximum weight that can be lifted in a single repetition) of 3 fundamental exercises (squat, deadlift, bench press) with low volume (i.e., few repetitions and sets) and RPE > 4. Additionally, a progressive periodization by increasing the volume by 5–10% every 2–3 weeks was prescribed, keeping controlled BP and HR before and after every training session. At the time of the physiotherapy follow-up visit (day 31), the patient only reported a left neck and hemi-thorax heaviness feeling during submaximal physical activity.

**Figure 4:**
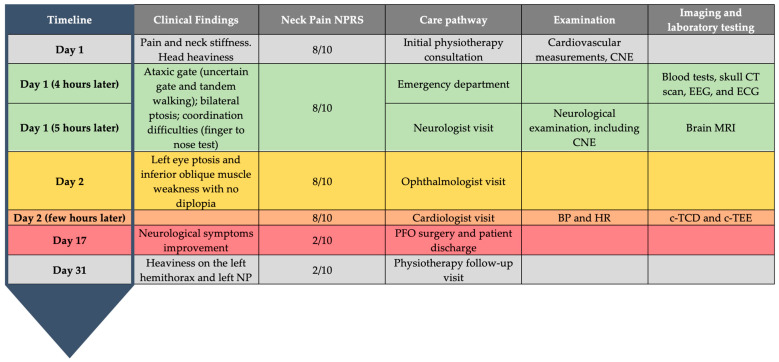
Timeline of the interventions, outcomes and follow-up. NP: neck pain; CNE: cranial nerve examination; CT: computerized tomography; MRI: magnetic resonance imaging; ECG: electrocardiography; BP: blood pressure; HR: heart rate; c-TCD: contrast transcranial doppler; c-TEE: contrast transesophageal echocardiogram; EEG: electroencephalogram; PFO: patent foramen ovale.

Although the mortality after surgical repair is very low and the recurrence of stroke or transient ischemic attack after trans-catheter PFO closure are unlikely (0–6%) [71,72,73,74], the patient was recommended to have another follow-up consultation within 30 days. For a detailed follow-up, outcomes and timeline of the management refer to Figure 4.

## 3. Discussion

Our case report illustrates the informed clinical decision-making process performed by a physiotherapist working in a direct access setting that led to triage a cardiovascular disease in a patient complaining of NP [75,76,77]. NP and head/orofacial symptoms have been already observed as the early manifestation of cardiovascular diseases (including PFO), vascular pathologies and blood flow limitations [8,64,65,78,79]. 

PFO is a congenital heart defect and consists of a physiological interatrial foramen during fetal life which is generally locked by fibrous adhesions among the septum primum and secundum during the earliest months of life [64]. The persistence of this foramen after birth is a benign anatomic anomaly most of the time; however, it is a potential cause of micro-emboli and minute blood clots which could pass the PFO, avoiding lungs’ lysis and then entering into the intracranial circulation causing stroke [65]. In young patients, it is estimated that more than 25% of persons suffering from a PFO had an increased risk of recurrent stroke. It has been shown that PFO is more frequently associated with cryptogenic stroke compared to patients with stroke of clear origin [62] and the general population [80,81]. Notably, the onset of stroke related to PFO can be sudden or subtle [62,82] and PFO is often an occasional finding in these patients, being a risk factor or a direct cause for stroke [62]. Several potential thromboembolic mechanisms have been proposed, but the main hypothesis relies on paradoxical embolism [65]. Accordingly, the inclusion of PFO in the hypothesis generation phase of the physiotherapy screening of referral process is a mainstay, as it is frequently associated with various serious pathological conditions [64]. Table 2 provides a PFO in-depth focus.

### Influence on Physiotherapy Clinical Practice 

Physiotherapy direct access is becoming a growing practice [84]; therefore, it is a priority and a professional responsibility to triage serious pathologies outside the scope of practice [13,15]. Among cardiovascular diseases, physiotherapists must be aware that not only vascular flow limitations—such as cervical arterial insufficiency, dissection or peripheral arterial disease (PAD) [21,23,24,25]—may mimic musculoskeletal pain, but also other conditions such as acute myocardial infarction [26] and PFO [70,79]. Thus, it is essential to possess the knowledge and the skills on how examine and interpret cardiovascular parameters [12,14,52]. In addition, physiotherapists must be prepared on the relevant concepts related to pathophysiology and differential diagnosis of cardiovascular pathological conditions, being able to appropriately consider findings from history and physical examination within their clinical reasoning [5,6,7]. History taking is essential to identify risk factors—such as HBP, thrombocytopenia and leukopenia—and subtle or transient neurological symptoms—such as head heaviness and balance disturbance—to make the best judgment on the probability of the presence of an unusual presentation [25,85]. HBP is the most common risk factor and stronger predictor of an increased risk of stroke [86]; notably, the majority of patients (>55%) do not recognize having HBP because it is commonly asymptomatic [87,88], further increasing the risk of lethal consequences [89]. A positive family history is common in patients with hypertension, with a likelihood of heritability estimated between 35% and 50% [90,91]. This genetic predisposition, along with environmental factors—such as high sodium and excessive alcohol intake, poor quality of sleep, sleep apnea and high mental stress—contribute to further increase the risk [92,93].

Although physiotherapists are not used to screen risk factors and blood pressure measurement in their daily practice [52], cardiovascular parameters play a key role in screening for the referral process [28,29,30,31,94] and HBP and high RHR must alarm the physiotherapist [95]. In Table 3 we provide a detailed description of the core skill set of testing required for the physiotherapy screening for referral.

Furthermore, physiotherapists must be aware that exercise-provoked acute heart attack increases by seven-fold the risk of death compared to sudden cardiac attack at rest [31]. The purpose of the cardiovascular parameters measurements, including during physical exertion, is to continue monitoring the cardiovascular hypothesis—under stressful conditions for the whole cardiovascular system—generated during the history. This provides support the clinicians’ decision making for a personalized and safe prescription of exercises or for a medical referral for further investigation [105,106,107,108]. 

## 4. Conclusions

To the best of the authors’ knowledge, this is the first documented case of PFO presenting as NP screened by a physiotherapist. Early detection and timely referral of unrecognized cardiovascular diseases ensures patient safety and appropriate medical management. Therefore, although these clinical presentations are rare, physiotherapists must be able to triage cardiovascular pathologies for safe patient management, ensuring an early referral when appropriate.

### Patient Perspective 

From the patient’s perspective, the timely and crucial physiotherapist’s decision making was a source of great gratitude. The patient was also satisfied of the communication skills of the physiotherapists in managing a delicate situation providing proper explanation and indication. He decided to continue his rehabilitation and return to sport under the physiotherapist’s supervision.

## Figures and Tables

**Table 1:** Blood pressure measurement during exercise.

Time (minutes)	Heart Rate (bpm)	Blood Pressure (mmHg)	RPE	Symptoms
0 (RHR and RBP were used as baseline pre-exercise measurement)	68	130/80	0	Neck pain and head heaviness
3	91	145/85	0	Neck pain and stiffness, head heaviness
6	96	160/95	5	Familiar NP onset
9	101	190/100	8	Sharp BP and NP increase, progressive unsteady gait
Immediately after exercise	97	180/95	7	Unsteady gait and NP
2 min after exercise	84	155/85	4	Unsteady gait and NP
4 min after exercise	72	130/80	2	Unsteady gait and NP

**Table 2:** Understanding the patent foramen ovale.

PFO: what is it?	The patent foramen ovale is a congenital heart defect, consisting of an opening in the inter-atrial septum resulting from incomplete coverage of the ostium secundum, by the septum secundum [65].
Major risk factors linked with PFO-referable strokes	PFO morphology and its size, young age, right-to-left shunt degree, atrial septal aneurysm, intrinsic coagulation–anticoagulation systems imbalance, other atrial abnormalities co-existence (as right atrial septal pouch, Eustachian valve and Chiari’s network) [62].
Clinical conditions frequently related with PFO	Decompression illness in scuba divers, platypnea–orthodeoxia syndrome, cryptogenic stroke, cerebral fat embolism syndrome, [70] obstructive sleep apnea (OSAS), migraine [64] with aura [65,79] and transient global amnesia [83].
PFO diagnostic procedures	The gold standard technique is represented by the contrast transesophageal echocardiography (c-TEE) but contrast transcranial doppler (c-TCD) and contrast-transthoracic echocardiography (c-TTE) are also utilized to establish the hemodynamic relevance and seriousness of the right-to-left shunting through a PFO [64,65,66].
PFO medical management	Trans-catheter closure consists of a percutaneous insertion of a device to close the interatrial septum and has been shown the less invasive surgical technique which allows patients to be discharged from hospital in 24 h [65].

**Table 3:** Core skill set testing for the physiotherapy screening for referral.

Upper motor neuron examination	The upper motor neuron examination includes assessment of reflexes, strength, coordination, muscle tone, muscle bulk and abnormal movements. Some of these may be detected through careful observation. However, specific maneuvers/tests (i.e., hyperreflexia, ankle clonus, finger rolling, pronator drift, Babinski sign and Hoffman’s reflex) help in the detection of abnormality. Abnormal findings prompt the suspicion of a central motor dysfunction in need urgent referral [15,61].
Cranial nerve examination	Cranial nerve examination is an integral part of a complete neurological examination and consists of assessing a subset of the twelve cranial nerves in order to identify any abnormalities in their function. Subtle or frank cranial nerve palsy may precede more serious pathologies mimicking neck pain or headache in early stages [24,96,97].
Peripheral neurological examination	The peripheral neurological examination consists of the assessment of the sensibility (including pain, temperature and light touch testing), the muscle strengths and deep tendon reflexes. Careful observation and specific tests are used to detect any abnormalities. The main purpose is to localize where in the peripheral nervous system the lesion is [15].
Blood pressure measurement	High systolic and diastolic pressure are the most common risk factors for cardiovascular pathologies and stroke [86]. These parameters are important to evaluate, in both during resting or physical activity, for the patient’ safety—preventing potentially fatal events—and the decision to refer and its urgency [12,14,47,52,58,98,99,100].
Heart rate measurement	Heart rate is an indirect parameter of coronary blood flow, myocardial performance and oxygen demand. It is a vital sign which can be easily monitored [47,101].
Blood pressure measurement during exercise	Together with electrocardiographic and heart rate monitoring, BP measurement during clinical exercise testing (GXT) must be considered in those patients at risk of masked hypertension. Exaggerated increases in systolic BP (SBP) and/or diastolic BP (DBP) during GXT are related with a 36% increased risk of cardiovascular events and mortality, and extended risk of hidden hypertension in normotensive subjects. Thus, exaggerated blood pressure response to exercise (EBPR) is a useful clinical test for diagnostic and prognostic purposes [54]. Both the American College of Sports Medicine (ACSM) and the American Heart Association (AHA) identify an increase of 8–12 mmHg of the SBP or 10 mmHg of the metabolic equivalent (MET; 3.5 mL∙kg^−1^∙min^−1^) as ‘‘normal’’ measurement. Additionally, both recommend GXT cessation when an increase of SBP > 210–250 mmHg and DBP > 115 mmHg is observed [102,103,104]. EBPR may help to unmask unmanifested cardiovascular diseases (e.g., coarctation of aorta), enhancing risk stratification and the sensitivity of stress studies (e.g., electrocardiogram), ensuring the early diagnosis and appropriate medical management [54]. The subjects had to continue the test until 90% of their adjusted-age maximal heart rate (HR) was obtained. SBP and DBP has to be recorded just before the exercise and during the last 30–45 s of every 3 min during the exercise [53]. At the end of the test, BP has to be recorded after three minutes of recovery with the patient in a sitting position. That is, based on the BP measurements, the test is evaluated as normal or exaggerated. The test must be stopped only at the occurrence of one of the following: achievement of more than 90% of the age-predicted maximal heart rate, severe fatigue or chest discomfort [54].

## Data Availability

Not applicable.

## Abbreviations

ACSM: American College of Sports Medicine; AHA: American Heart Association; BP: blood pressure; CN: cranial nerve; c-TCD: contrast transcranial doppler; c-TEE: contrast transesophageal echocardiogram; GXT: clinical exercise testing; HBP: High blood pressure; HR: heart rate; NP: neck pain; NPRS: numeric pain rating scale; PAD: peripheral arterial disease; PFO: patent foramen ovale; RBP: resting blood pressure; RHR: resting heart rate; RM: maximum repetition; RPE: rate of perceived exertion.
